# Synthesis of novel ICIE16/BSG and ICIE16/BSG-NITRI bioglasses and description of ionic release kinetics upon immersion in SBF fluid: Effect of nitridation

**DOI:** 10.1016/j.dib.2015.11.026

**Published:** 2015-11-18

**Authors:** Felipe Orgaz, Daniel Amat, Olga Szycht, Aleksandra Dzika, Flora Barba, José Becerra, Leonor Santos-Ruiz

**Affiliations:** a Instituto de Cerámica y Vidrio, Consejo Superior de Investigaciones Científicas (ICV-CSIC), c/Kelsen no. 5, 28049 Madrid, Spain; b Universidad de Málaga, Departamento de Anatomía y Medicina Legal, Facultad de Medicina, Campus de Teatinos, 29071 Málaga, Spain; c Centro de Investigación Biomédica en Red, Bioingeniería, Biomateriales y Nanomedicina (CIBER-BBN), Instituto de Salud Carlos III, c/Monforte de Lemos 3-5, Pabellón 11, Planta 0, 28029 Madrid, Spain; d Universidad de Málaga & IBIMA, Departamento de Biología Celular, Genética y Fisiología, Facultad de Ciencias, Campus de Teatinos, 29071 Málaga, Spain; e BIONAND-Universidad de Málaga, c/Severo Ochoa 35, Campanillas, 29590 Málaga, Spain

**Keywords:** Biomaterials, Bioglass, Simulated body fluid, Degradability, Biomaterial resorption, Bone repair

## Abstract

A novel bioactive glass scaffold ICIE16/BSG has been prepared from a mixture of two different melt-derived glasses: a silicate bioglass (ICIE16) and a borosilicate bioglass (BSG). Combined processing techniques (gel casting and foam replication) were used to form three-dimensional, interconnected porous monolith scaffolds (Orgaz et al., 2016) [1]. They were then nitrided with a hot ammonia flow as described in (Aleixandre et al., 1973) [3] and (Nieto, 1984) [4] to synthesize the ICIE16/BSG-NITRI bioglass (Orgaz et al., 2016) [1]. Herein we present a flow chart summarizing the forming process, plus images of the resulting scaffold after sintering and drying. Bioactivity was characterized in vitro by immersion in simulated body fluid (SBF) for up to seven days. Data of ionic release kinetics upon SBF immersion are presented.

Specifications Table

**Table:** 

Subject area	*Chemistry, Biology*
More specific subject area	*Bone tissue engineering*
Type of data	*Text file, flow chart, graph*
How data was acquired	*Dissecting microscope, Plasma emission spectroscopy*
Data format	*Raw, analyzed,*
Experimental factors	
Experimental features	*Bioglasses synthesized as in**[1]**were photographed with a dissecting microscope to show 3D porous architecture. Then, they were immersed in SBF for 7 days and released ions and soluble species were evaluated by Plasma emission spectroscopy (ICP – OES, Thermo Jarrell Ash IRIS)*
Data source location	
Data accessibility	*The data is available with this article and is related to*[1]

Value of the data

• To illustrate the manufacturing of a novel glass–glass composite scaffold with biomedical potential. This manufacturing process could be used by other authors to prepare other glass–glass composites.
• To show the hierarchical porous structure of the scaffolds, needed in biomaterials with intended use in bone repair.
• To show the release kinetics of cell-influencing ions Calcium, Phosphorous, Silicon, Potassium and Boron upon immersion in SBF.
• To show how the above-mentioned release kinetics is influenced by nitridation. After these data, nitridation could be used by other material scientists interested in modifying the degradability and ion-release of their bioglasses.

## 1 Data

**Fig. 1:**
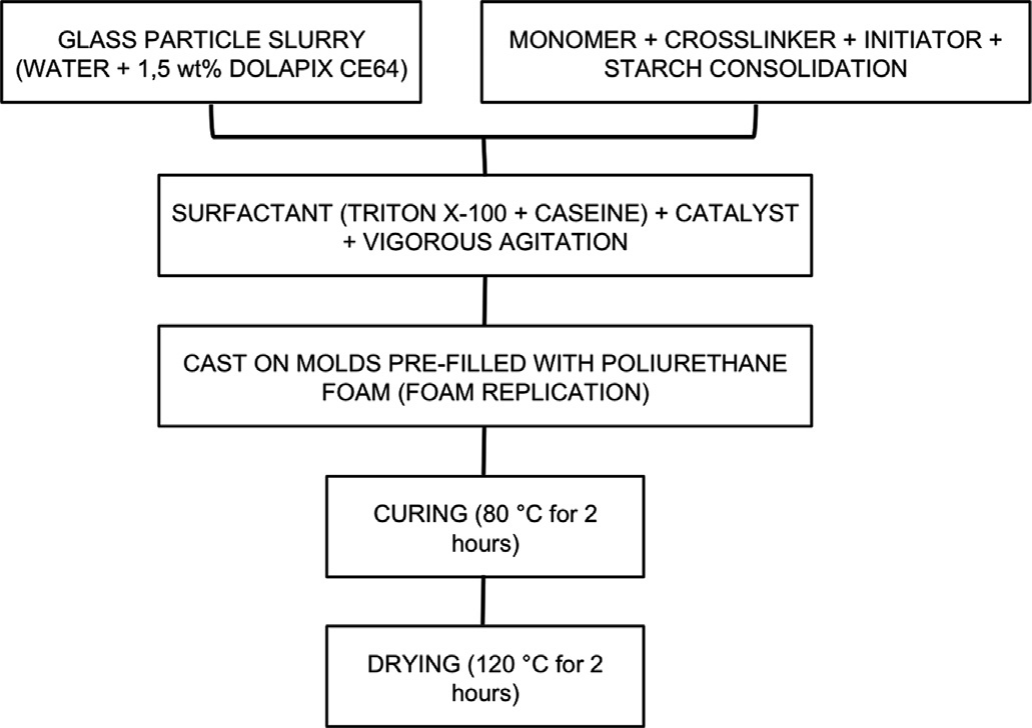
Flow Chart of the sintering process of ICIE16/BSG bioglasses.

**Fig. 2:**
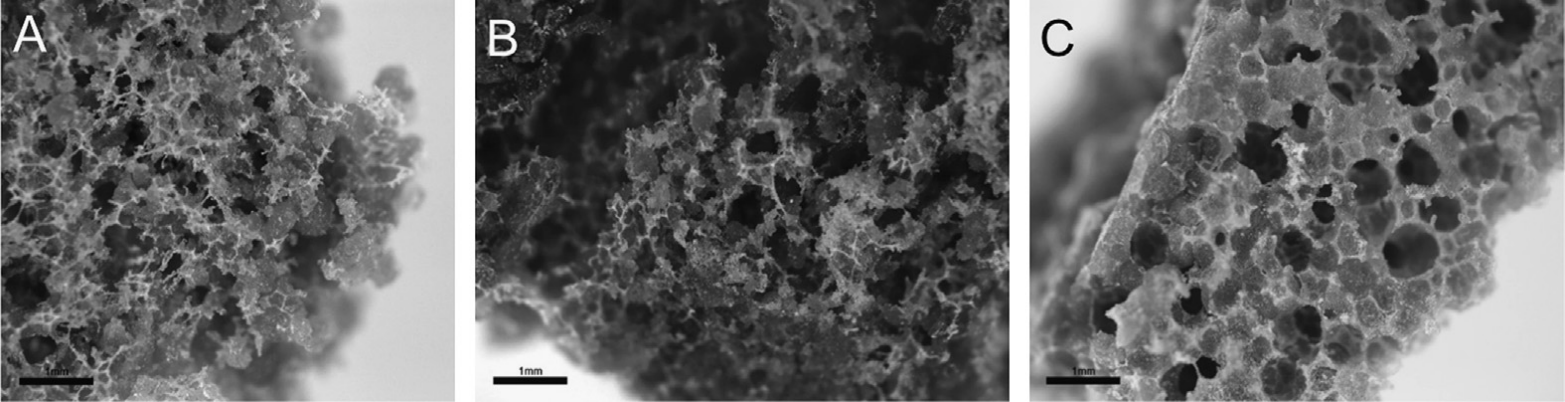
Images showing the ICIE16 (A), ICIE16/BSG (B) and ICIE16/BSG-NITRI (C) bioglasses. A porous architecture with porous of different sizes can be observed in all of them. Scale Bar: 1 mm.

Fig. 1 summarizes the sequential steps that make up the processing method of ICIE16/BSG bioglasses. The resulting porous 3D scaffolds can be observed in Fig. 2.

**Fig. 3:**
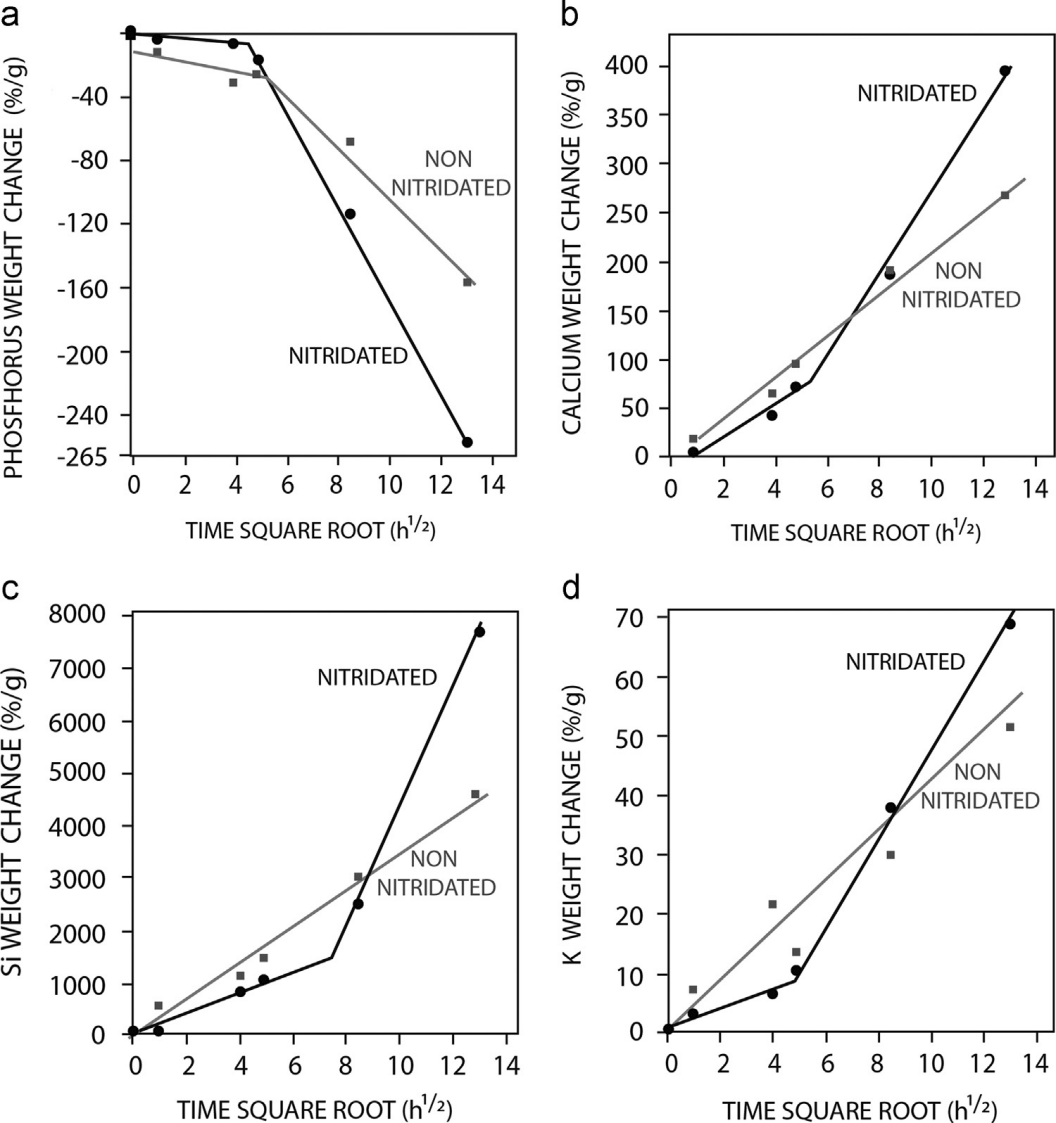
Ion changes (% weight) of Calcium, Phosphorous, Silicon and Potassium in nitrided and non-nitrided ICIE16/BSG scaffolds upon immersion in SBF.

Fig. 3 collects the changes in Calcium, Phosphorous, Silicon and Potassium. As observed, the concentration of phosphorous in SBF decreases with time, while calcium concentration increases. The concentration of Si and K ions also increases in the SBF over time.

**Fig. 4:**
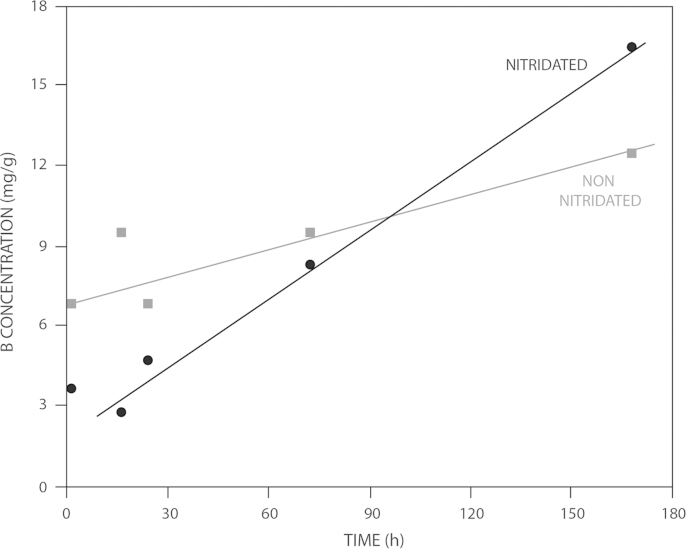
Boron ion changes in SBF after immersion of non-nitrided ICIE16/BSG and nitrided ICIE16/BSG-NITRI bioglasses.

Fig. 4 illustrates changes in Boron content in the SBF after scaffold immersion. At low immersion times (<90 h) the amount of B released was lower for nitrided glasses. At longer times the trend was inverted.

## 2 Experimental design, materials and methods

Porous glass scaffolds ICIE16/BSG and ICIE16/BSG-NITRI were synthesized as described in [1].

Images of the bioglasses were taken with a dissecting microscope (Nikon AZ-100) equipped with a digital camera (Nikon Digital Sight DS-5Mc), operated by Nis-Elements software

*The porous glass scaffolds were immersed in a simulated body fluid (SBF) solution at 37 °C (prepared according to Kokubo et al.* [2] *for up to 168 h (7 days). A ratio of 1 g scaffold to 100 ml of SBF was employed in all the experiments. Calcium, Phosphorous, Silicon, Potassium and Boron ions released into the SBF solution were measured daily by Plasma emission spectroscopy (ICP – OES, Thermo Jarrell Ash IRIS). Weight changes (Δ*W*) were calculated as* Δ*Wt=100·(W0−Wt)/W0*

*where W0 is the initial mass and Wt is the mass at time t*.

*Weight changes data were plotted against the square root of time for Calcium, Phosphorous, Silicon, Potassium, and against time for Boron.*

## References

[1] Orgaz F., Dzika A., Szycht O., Amat D, Barba F, Becerra J., Santos-Ruiz L. (2016). Surface nitridation improves bone cell response to melt-derived bioactive borosilicate glass scaffolds. Acta Biomater..

[2] Kokubo T., Kushitani H., Sakka S., Kitsugi T., Yamamuro T. (1990). Solutions able to reproduce in vivo surface–structure changes in bioactive glass-ceramic A-W. J. Biomed. Mater. Res..

[3] Aleixandre V., Fernández Navarro J., Oteo J.L. (1973). The incorporation of nitrogen to alkali borate glasses at different temperatures. Bol. Soc. Esp. Ceram. Vidr..

[4] M.I. Nieto, Reactions of ammonia with glasses containing B_2_O_3_ as network former, 1984

